# Tooth Wash

**Published:** 1856-10

**Authors:** 


TOOTH WASH.—The safest, cheapest, most universally accessible, and most efficient, is a piece of White Soap, with a moderately stiff tooth brush, every morning. In addition, immediately after each meal, use simple tepid water, with a brush not so stiff, use it slowly, with a perpendicular twist, so as to remove particles of food more thoroughly from between the teeth. At the same time, twist the brush horizontally across the back part of the tongue. In this way, the smell of the food on the breath of a recent meal is at once removed. It is a bad plan to defer teeth cleaning from supper until bed time, as it only gives the accretions several hours to work their mischief.

